# Supplementary material for: Human papilloma virus vaccination uptake and associated factors among adolescent girls in Merab Abaya district, Gamo zone, Southern Ethiopia: Mixed methods
Source: PLoS One. 2025 Sep 5;20(9):e0330760. doi: 10.1371/journal.pone.0330760 (PMC12412932; doi:10.1371/journal.pone.0330760)
Supplement: Attachment — Submitted filename: Reviewers comments(HG).docx [file pone.0330760.s001.docx]

# Key Comments & Suggested Revisions:

**1. Abstract**

- **Strengths:**
- Clearly outlines the study’s background, objective, methods, results, and conclusion.
- Highlights the main statistical findings concisely.
- **Areas for Improvement:**
- **Language & Grammar:** The abstract contains multiple grammatical and structural errors. Needs proofreading for better readability.
- **Methodology Clarification:** The mention of “two-stage sampling” and “purposive sampling” should be slightly elaborated for clarity.
- **Qualitative Findings Integration:** The abstract heavily focuses on quantitative results. Briefly summarizing key qualitative barriers would strengthen the abstract.
- **Suggestion:**
- Improve clarity and grammar.
- Include qualitative findings concisely.
- Clarify key methodological aspects.

**2. Introduction**

- **Strengths:**
- Provides a strong rationale for the study with a global and regional perspective on HPV vaccination.
- Discusses the burden of cervical cancer and vaccination strategies.
- **Areas for Improvement:**
- **Ethiopian Context:** Needs more statistics specific to Ethiopia’s HPV vaccination program coverage.
- **Study Gap & Justification:** The gap in school vs. community-based adolescent girls is a key contribution but should be emphasized more clearly.
- **Policy Relevance:** How can findings inform Ethiopia’s national HPV vaccination strategy?
- **Suggestion:**
- Add national data on HPV vaccination rates.
- Explicitly state how this study fills the research gap.
- Connect findings to national health policy implications.

**3. Methods**

- **Strengths:**
- Provides a structured explanation of study design, population, and sampling.
- The two-stage sampling approach and use of mixed methods are appropriate.
- **Areas for Improvement:**
- **Study Design Justification:** The choice of a mixed-methods design should be explicitly justified.
- **Qualitative Methods Weakness:**
  - Lack of details on **how qualitative participants were selected** and **how data saturation was determined**.
  - The qualitative component is not well-integrated with quantitative findings.
- **Statistical Analysis Clarity:**
  - The rationale for using logistic regression should be briefly explained.
  - Define statistical assumptions (e.g., multicollinearity check, goodness-of-fit test).
- **Ethical Considerations:**
  - Ethical approval and informed consent are mentioned, but there is **no discussion on how participant confidentiality was maintained**.
  - **Parental consent** for minors should be explicitly stated.
- **Suggestion:**
- Clarify why a mixed-methods approach was chosen.
- Improve details on qualitative data collection and analysis.
- Provide more details on statistical validity checks.
- Strengthen the ethical considerations section.

**4. Results**

- **Strengths:**
- Clearly presents both descriptive and inferential statistics.
- Tables and figures effectively summarize key findings.
- **Areas for Improvement:**
- **Inconsistent Data Presentation:**
  - The **qualitative findings are summarized separately**, making it difficult to integrate with the quantitative results.
  - The qualitative barriers should be incorporated **within the quantitative discussion** to create a cohesive narrative.
- **Confidence Intervals & p-values:**
  - Some tables lack **confidence intervals for adjusted odds ratios**.
  - Ensure all variables in multivariate models have their respective p-values reported.
- **Missing Effect Sizes for Key Findings:**
  - Instead of just stating associations, include an interpretation of effect sizes (e.g., **"Family support increases the odds of HPV uptake by sevenfold"**).

**Suggestion:**

- Integrate qualitative and quantitative findings for a more coherent discussion.
- Ensure consistent reporting of confidence intervals and p-values.
- Interpret effect sizes meaningfully.

**5. Discussion**

- **Strengths:**
- Findings are compared with other studies.
- Practical recommendations are given.
- **Areas for Improvement:**
- **Thematic Integration Weakness:**
  - The discussion mostly focuses on quantitative results, with **minimal integration of qualitative themes**.
  - The qualitative findings should be used to **explain why** certain factors influence HPV uptake.
- **Study Limitations Section is Weak:**
  - It should include **potential biases** (e.g., self-reporting bias, recall bias).
  - Acknowledge limitations in the generalizability of findings.
- **Policy & Practice Implications Need Strengthening:**
  - How can these findings inform vaccination strategies in Ethiopia?
  - What targeted interventions can improve uptake?

**Suggestion:**

- Improve qualitative-quantitative integration.
- Expand the limitations section.
- Strengthen the practical and policy implications.

**6. Conclusion**

- **Strengths:**
- Summarizes key findings concisely.
- Provides recommendations.
- **Areas for Improvement:**
- Should **explicitly connect findings to policy recommendations**.
- Needs to **acknowledge study limitations** more clearly.

**Suggestion:**

- Strengthen connection to policy and public health strategies.
- Mention study limitations.

**Language & Formatting Issues**

**Major concerns:**

- **Numerous grammatical and typographical errors** throughout the manuscript.
- Sentence structure and wording need improvement for clarity and readability.

**Suggestion:**

- **Professional proofreading & language editing** is strongly recommended.
- Consider **restructuring certain paragraphs** for better flow.

**Key Actions Needed Before Resubmission:**

1. **Improve language clarity & proofreading.**
2. **Enhance integration of qualitative and quantitative findings.**
3. **Clarify statistical methodology and reporting.**
4. **Strengthen ethical considerations, limitations, and policy implications.**
5. **Improve discussion depth and coherence.**
