# Supplementary material for: Human papilloma virus vaccination uptake and associated factors among adolescent girls in Merab Abaya district, Gamo zone, Southern Ethiopia: Mixed methods
Source: PLoS One. 2025 Sep 5;20(9):e0330760. doi: 10.1371/journal.pone.0330760 (PMC12412932; doi:10.1371/journal.pone.0330760)
Supplement: Attachment — Submitted filename: Response to academic ed and rev HPV.docx [file pone.0330760.s002.docx]

**Response to academic editor and reviewers	May 30, 2025**

**To: Morufu Olalekan Raimi, Ph.D (Academic Editor, PLOS ONE)**

**PONE-D-25-02990**

**Human Papilloma Virus Vaccination uptake and Associated Factors among Adolescent Girls in Merab Abaya District, Gamo Zone, Southern Ethiopia: Mixed Methods**

Dear Academic Editor,

Thank you for considering our manuscript and for arranging for it to be reviewed by reviewers. We have tried to address your comments and the comments / suggestions from the reviewers. We have highlighted the changes within the manuscript please find for your kind consideration. In the Response to Reviewers, we copy each of the *comments* or *suggestions* and provide the response hereunder with by number determined as **Author response**. We also provide a marked-up copy of the manuscript that highlights changes made to the original version and this is uploaded as a separate file labeled “*Revised Manuscript with Track Changes*”. Finally, we provide a cleaned version of the revised manuscript without tracked changes and this is uploaded as a separate file labeled *'Manuscript*'.

We have been carefully through the peer review and have revised our paper accordingly. We feel that the paper is much improved as a result of this peer review process, and thank you for taking it to this stage. We hope we have satisfactorily addressed all the comments and hope that our paper may now be suitable for publication in your journal and we keep in touch that we are waiting yours feedback also. We hold ourselves at your entire disposition for any further information or other changes you might require.

Sincerely yours!

Tamirat Mathewos Milkano (Corresponding author)

**Response to academic editor**

1. Journal Requirements: Please ensure that your manuscript meets PLOS ONE's style requirements, including those for file naming

**Author response:** Thanks for reviewing our paper deeply. We reviewed our manuscript and revised it as to meet the PLOS ONE's style requirements.

**2.** We note that your Data Availability Statement is currently as follows: All relevant data are within the papers and its supporting information files. But the qualitative parts of the study, the participants were not consented to share their audio.

**Author response:** Thanks for reviewing our paper deeply. For the qualitative data consent were took from participants before data collection and after their agreement we have started collecting data, the audio agreement was also in our hand, ‘’the concept saying the participants are not consented to share their audio’’ means no consent were taken from the participants during data collection about sharing their audio for publication issue, if their audio is mandatory and needed for publication we will try to find them and share it during publication process.

Other data sets are available in time during publication and in any time.

3. One of the noted authors is a group or consortium. In addition to naming the author group.

**Author response:** Thanks for reviewing our paper deeply and giving constructive comments. We have reviewed and modified the authors with their roles and affiliation manuscript.

**Additional Editor Comments:**

**1. Language and Grammar:** The manuscript contains numerous grammatical and structural errors, which hinder readability. Professional proofreading and language editing are strongly recommended.

**Author response:** Thanks for deeply reviewing of our paper and giving recommendations to be modified and corrected. Our manuscript undergone proof reading and English grammar check by professionals and some online websites, we also acknowledge the one who helped us in proof reading of our manuscript document and we also ensure that the manuscript undergoes through English language editing and document formatting to improve its clarity and readability.

**2. Methodological Clarity:** Please provide a clearer justification for the mixed-methods design, elaborate on sampling techniques, and ensure transparency in statistical analyses. The qualitative component, in particular, requires more detail regarding participant selection and data saturation.

**Author response:** Thanks for deeply reviewing of our paper and giving recommendations to be modified and corrected. It is elaborated on both clean and track change manuscript for both.

**3. Integration of Findings:** The qualitative and quantitative findings are currently presented separately, leading to a lack of cohesion. A more integrated discussion would enhance the manuscript’s narrative and impact.

**Author response:** Thanks for deeply reviewing of our paper and we appreciate you taking the time to review our manuscript. It is elaborated on both clean and track change manuscript for qualitative and quantitative findings, by keeping cohesion and integrated discussion was done for enhancing the impact of the manuscript.

**4. Ethical Considerations:** While ethical approval and informed consent are mentioned, the section needs to be expanded to include details on confidentiality and parental consent for minors.

**Author response:** Thanks for deeply reviewing of our paper and giving recommendations to be modified and corrected. In any situation of research work the ethical consideration were appropriately applied, considering minors in time of research work was also applied by making appropriate communication with their parents. Verbal consent was undertaking for the issue. Major ethical principles like beneficence, non-maleficence, autonomy, and justice were followed in any situation of research work.

**5. Policy and Practical Implications:** The discussion and conclusion should more explicitly connect the findings to Ethiopia’s national HPV vaccination strategy and propose targeted interventions to improve uptake.

**Author response:** Thanks for deeply reviewing of our paper and we appreciate you taking the time to review our manuscript. We have considered all the policy and strategies currently working in Ethiopia for HPV vaccination implementation and indication. All the discussion conclusion with recommendations were done based on current policy of Ethiopia.

**Reviewers' comments to the Author**

1. Is the manuscript technically sound, and do the data support the conclusions? The manuscript must describe a technically sound piece of scientific research with data that supports the conclusions. Experiments must have been conducted rigorously, with appropriate controls, replication, and sample sizes. The conclusions must be drawn appropriately based on the data presented.

**Reviewer #1: Partly:** **Author response:** Thank you. We appreciate you taking the time to review our manuscript. We have considered all your implication and tried to make our manuscript technically sound and rigorous work conducted in any step of the work for publication and for valid scientific finding, appropriate and relevant conclusion was drawn from the research work.

2. Has the statistical analysis been performed appropriately and rigorously?

Reviewer #1: Yes

**Author response:** Thank you. We appreciate you taking the time to review our manuscript

3. Have the authors made all data underlying the findings in their manuscript fully available?

The [PLOS Data policy](http://track.editorialmanager.com/CL0/http:%2F%2Fwww.plosone.org%2Fstatic%2Fpolicies.action%23sharing/1/010f01953663bc1c-1b728dec-6154-49aa-9113-330c96417392-000000/4gkvUK7TP362_5mnlRGMem8Qz1DIsjR2DDxz31yPM88=199) requires authors to make all data underlying the findings described in their manuscript fully available without restriction, with rare exception (please refer to the Data Availability Statement in the manuscript PDF file). The data should be provided as part of the manuscript or its supporting information, or deposited to a public repository. For example, in addition to summary statistics, the data points behind means, medians and variance measures should be available. If there are restrictions on publicly sharing data. e.g., participant privacy or use of data from a third party—those must be specified.

Reviewer #1: Yes

**Author response:** Thank you. We appreciate you taking the time to review our manuscript

4. Is the manuscript presented in an intelligible fashion and written in standard English?
PLOS ONE does not copyedit accepted manuscripts, so the language in submitted articles must be clear, correct, and unambiguous. Any typographical or grammatical errors should be corrected at revision, so please note any specific errors here.

Reviewer #1: Yes

**Author response:** Thank you. We appreciate you taking the time to review our manuscript. We have deeply edited the writing to make it in intelligible fashion and English language editing was intensively done by software’s and website.

**5. Review Comments to the Author**

Please use the space provided to explain your answers to the questions above. You may also include additional comments for the author, including concerns about dual publication, research ethics, or publication ethics. (Please upload your review as an attachment if it exceeds 20,000 characters)

**Author response:** Thank you. We appreciate you taking the time to review our manuscript. There is no dual publication were done. Ethical issues were fully considered by using basic research ethics principles.

**Reviewer #1: Key Comments & Suggested Revisions:**

**1. Abstract**

**Strengths:** Clearly outlines the expected contents

**Author response:** Thank you. We appreciate you taking the time to review our manuscript.

**Areas for Improvement:** Improve clarity and grammar, include qualitative findings concisely, Clarify key methodological aspects

**Author response:** Thank you. We appreciate you taking the time to review our manuscript. All the comments were modified in both manuscript clean and with track change

**2. Introduction**

**Author response:** We appreciate the reviewer for taking the time to review our manuscript and for giving the constructive comments and suggestions as you provided as strength.

**Areas for Improvement and suggestions:** Improve on more statistic data on Ethiopian context, appropriate justification on gaps, support policy relevance, add national data on HPV vaccination rates and connection of finding to national health policy.

**Author response:** Thank you. We appreciate you taking the time to review our manuscript. All the comments were modified in both manuscript clean and with track change.

Authors tried to incorporate and correct all the suggestions and areas needed to be improved. In the content suggesting that add more statistical data from Ethiopian context: there are less amount of study done which are relevant for our study were included. All the needed and relevant justifications describing gaps were incorporated in the manuscript in both clean and with track change. Justification supporting the policy relevance and connecting finding to the national health policy were also modified in manuscript in both clean and track changed.

**3. Methods**

**Author response:** We appreciate the reviewer for taking the time to review our manuscript and for giving the constructive comments and suggestions as you provided as strength.

**Areas for Improvement and suggestions:** Improve on more on area of study design in both quantitative and qualitative part, ethical consideration, validity checking mechanism

**Author response:** Thank you. We appreciate you taking the time to review our manuscript. All the comments were modified in both manuscript clean and with track change.

Authors tried and corrected all comments and suggestions to be improved in methodology part.

**Study Design Justification:** The reason for using mixed method in the study were there were findings that support the uptake of HPV vaccine were affected by cultural issues and other factors which needs in-depth interview and supporting the quantitative data.

**Qualitative Methods Weakness:** Integration of qualitative with quantitative is done in manuscript. The data saturation is happened at participant 8 from minimum expected sample size to be needed which is 16.

**Statistical Analysis Clarity:** The reason for using logistic regression analysis for analysis is most of the studies related to the title are done in logistic analysis and the concept mainly done by making it dichotomous.

Multicolinearity test is method checking two or more independent variables are related with each other and can bring effect on outcome variable. The test was done by checking variance inflation factor and tolerance teste in SPSS. The GoF or goodness of fit test is done by Hosmer lemshow test and the variables in significant range or <0.05 took as good model fit.

**Ethical Considerations:** all type of ethical principles were considered and conducted in different steps of research process, regarding minors’ discussion between their parents and if they were in school their respective school director was conducted regarding the research process, maintaining confidentiality were taken in any phase of research which was also done by common agreement among participants and researchers.

**Suggestions: *Improve details on qualitative data collection and analysis:*** The interview was conducted by the principal investigator. A semi-structured interview guide was prepared and used in both the in-depth interview and focus group discussion. The principal investigator was taken video-based lectures to upgrade his capacity on how to approach participants, how to handle them during interviews, how to probe, and how to ask sensitive questions. FGDs’ participants were appointed one week before the meeting for discussion, and participants consisting of (health professionals, and teachers).

The average time used for IDI and FGD were 24:30 and 1:11:35 minutes respectively. Study participants were informed about the purpose of the study and what the research is all about before recruiting them to the study at first contact. Then the interview at quiet places with maximum privacy had been chosen for both IDIs and FGDs. On the other hand, issues of safety for both the principal investigator and the study participants was the main considerations while selecting that place. The topic guide addressed all issues related to barriers with human Papilloma virus uptake and other issues relevant to the objectives of the study. The principal investigator had conducted the discussions, while one assistance co-facilitator had assigned during the FGDs to manage discussion time and facilitate recording. IDI and FGD were guided by an experienced person fluent in the local language (Gammotho) and English with the researcher. The interview had been recorded on tapes, which were later transcribed. Detailed field notes had been taken.

**Provide more details on statistical validity checks.** Statistical validity of the tool initially was done by expert and additional validity test was also done. To maintain the internal consistency of the instrument, the items' Cronbach's alpha coefficient was calculated. For qualitative data, one research assistance having qualitative data collection experience and principal investigators using interview guides (open-ended) was used. The data normality was done by Shapiro wilk test.

**4. Results:**

**Author response:** We appreciate the reviewer for taking the time to review our manuscript and for giving the constructive comments and suggestions as you provided as strength.

**Areas for Improvement and suggestions:** Improve on more on area of data Presentation, CI and P-values, missing effect sizes for key findings and its interpretation.

**Author response:** Thank you. We appreciate you taking the time to review our manuscript. All the comments were modified in both manuscript clean and with track change.

Authors tried and corrected all comments and suggestions to be improved in result part.

**Inconsistent Data Presentation:** The presentation of qualitative part in different were, since it is reporting and the result reporting needed to be done by itself. The discussion part was done by merging both qualitative and quantitative together, analysis generally done by quantitative followed by qualitative approach, integration of both quantitative and qualitative findings for a more coherent discussion and all needed modifications were done in both manuscripts.

**Confidence Intervals & p-values:** variables in tables which lack confidence interval were modified in manuscript both in clean and with track changes. The reason for not putting P- value is mainly due to that they are not significantly associated. Writing P-vale and confidence interval with effect size were mainly done factor which affects the uptake of HPV- vaccination.

**Missing Effect Sizes for Key Findings:** Its description was mainly done in discussion, meaningful interpretation was also done and additionally needed modifications were done in both clean and track change manuscripts**.**

**5. Discussion:**

**Author response:** We appreciate the reviewer for taking the time to review our manuscript and for giving the constructive comments and suggestions as you provided as strength in discussion part and all other parts of our manuscript.

**Areas for Improvement and suggestions:** Improve on more on area of Thematic Integration, Study Limitations, Policy & Practice Implications and its qualitative-quantitative integration.

**Author response:** Thank you. We appreciate you taking the time to review our manuscript. All the comments were modified in both manuscripts. Authors tried and corrected all comments and suggestions to be improved in discussion part.

**Thematic Integration Weakness:** Appropriate Integration of all the selected themes were appropriately done both clean and track change type manuscripts. In the manuscripts it deeply focused why certain factors influence HPV uptake in the study area among selected participants

**Study Limitations Section is Weak:** All the needed and important limitations or weakness were described in both manuscripts.

**Policy & Practice Implications Need Strengthening:** Policy and practice implications needs to be described in the study were mainly focus on current health policy system of Ethiopia and it considered about its implementation in expected period. It is more elaborated in manuscript.

**6. Conclusion**

**Author response:** We appreciate the reviewer for taking the time to review our manuscript and for giving the constructive comments and suggestions as you provided as strength in conclusion part and all other parts of our manuscript.

**Areas for Improvement and suggestions:** Improve more on area of connecting findings to the policy, more clearly acknowledge study limitations.

**Author response:** Thank you. We appreciate you taking the time to review our manuscript. All the comments were modified in both manuscripts. Authors tried and corrected all comments and suggestions to be improved in conclusion part. Connecting findings current and working healthy policy of Ethiopia were done and edited in the manuscript. The limitations of the study were described and written in manuscript also. Grammatical errors and major suggestions were also considered and authors tried to correct them in both manuscript.

7. PLOS authors have the option to publish the peer review history of their article ([what does this mean?](https://track.editorialmanager.com/CL0/https:%2F%2Fjournals.plos.org%2Fplosone%2Fs%2Feditorial-and-peer-review-process%23loc-peer-review-history/1/010f01943945507f-90f16986-018d-41fc-872b-8bc93c362a78-000000/QnEdzbPws-VffUjKhLkmWXwoh4PAMl5taqb10cN0-cU=192)). If published, this will include your full peer review and any attached files.
If you choose “no”, your identity will remain anonymous but your review may still be made public.
**Do you want your identity to be public for this peer review?** For information about this choice, including consent withdrawal, please see our [Privacy Policy](https://track.editorialmanager.com/CL0/https:%2F%2Fwww.plos.org%2Fprivacy-policy/1/010f01943945507f-90f16986-018d-41fc-872b-8bc93c362a78-000000/x-vKCSnLcgD353RjlycqV_VoK4IETpMlJBLn992ROKI=192).

Reviewer #3: No

**Author response:** Thank you. We appreciate you taking the time to review our manuscript.
