# Supplementary material for: Human papilloma virus vaccination uptake and associated factors among adolescent girls in Merab Abaya district, Gamo zone, Southern Ethiopia: Mixed methods
Source: PLoS One. 2025 Sep 5;20(9):e0330760. doi: 10.1371/journal.pone.0330760 (PMC12412932; doi:10.1371/journal.pone.0330760)
Supplement: Attachment — Submitted filename: 2nd Response to academic ed and rev HPV.docx [file pone.0330760.s003.docx]

**Response to academic editor and reviewers July 28, 2025**

**To: Morufu Olalekan Raimi, Ph.D(Academic Editor, PLOS ONE)**

**PONE-D-25-02990**

**Human Papilloma Virus Vaccination uptake and Associated Factors among Adolescent Girls in Merab Abaya District, Gamo Zone, Southern Ethiopia: Mixed Methods**

Dear Academic Editor,

Thank you for considering our manuscript and for arranging it to be reviewed by reviewers. We have tried to address your comments and there is no attachment describing about reviewers point in corresponding author email and website. We have highlighted the changes within the manuscript by considering comments. The response to comments were hereunder determined as **Author response**.We also provide a marked-up copy of the manuscript that highlights changes made to the original version and this is uploaded as a separate file labeled “*Revised Manuscript with Track Changes*”. Finally, we provide a cleaned version of the revised manuscript without track changes and this is uploaded as a separate file labeled *'Manuscript*'.

We have been carefully through the peer review and have revised our paper accordingly. We feel that the paper is much improved as a result of this peer review process, and thank you for taking it to this stage. We hope we have satisfactorily addressed all the comments and hope that our paper may now be suitable for publication in your journal and we keep in touch that we are waiting yours feedback also. We hold ourselves at your entire disposition for any further information or other changes you might require.

Sincerely yours!

Tamirat Mathewos Milkano (Corresponding author)

**Response to academic editor**

***1. Journal Requirements****:*Please review your reference list to ensure that it is complete and correct. If you have cited papers that have been retracted, please include the rationale for doing so in the manuscript text, or remove these references and replace them with relevant current references. Any changes to the reference list should be mentioned in the rebuttal letter that accompanies your revised manuscript. If you need to cite a retracted article, indicate the article’s retracted status in the References list and also include a citation and full reference for the retraction notice.

**Author response:**Thanks for reviewing our research work deeply. All the references were reviewed and no change have been made, retracted paper was not used in our work.

**Additional Editor Comments:**

After reviewing the manuscript and the authors' responses to the reviewers' comments, here is my assessment and recommendation:

**Strengths of the Manuscript:**

**1**. The mixed-methods approach (quantitative and qualitative) provides a robust analysis of HPV vaccine uptake and associated factors, enhancing the depth of understanding.

**Author response:** Thanks for reviewing our research work deeply. The authors want to appreciate the editor for giving constructive comments and suggestions on the area of relevance of the design and its implication on selected title.

**2.** The study aims to assess HPV vaccine uptake among adolescent girls in a specific region, addressing a gap in existing literature.

**Author response:** Thanks for reviewing our research work deeply andgiving constructive comments and suggestions.

**3.**The sampling technique, data collection, and analysis are well-described, ensuring reproducibility.

**Author response:**Thanks,from bottom of our heart for reviewing our research work deeply andgiving constructive comments and suggestions.

**4.** The study identifies key factors influencing vaccine uptake (knowledge, attitude, health worker recommendations, family support) and barriers (mistrust, irregular provision, lack of information).

**Author response:** Thanks, from bottom of our heart for reviewing our research work deeply andgiving constructive comments and suggestions.

**5.** Ethical approval and consent procedures are documented, and confidentiality measures are highlighted.

**Author response:** Thanks, from bottom of our heart for reviewing our paper deeply andgiving constructive comments and suggestions.

**Areas Addressed in Revisions:**

• The authors state that the manuscript underwent professional proofreading and language editing.

• Additional details on sampling techniques, qualitative data saturation, and statistical validity checks were provided.

• The discussion now better integrates qualitative and quantitative results.
• Expanded details on confidentiality and parental consent for minors were included.
• The discussion now explicitly connects findings to Ethiopia’s national HPV vaccination strategy.

**Author response:** Thanks, from bottom of our heart for reviewing our paper deeply andgiving constructive comments and suggestions. The authors also thank again the editor for clearly reverifying what we have addressed the correction of first review from reviewers and editor.

**Remaining Concerns:**

1. The qualitative audio data are not shared due to consent issues, which may limit transparency. However, the authors offer to provide it if mandatory.

**Author response:** Thanks, from bottom of our heart for reviewing our paper deeply andgiving constructive comments and suggestions. The authors ensure that we will share the audio data in any sharing method if it is mandatory.

2. The study’s focus on one district may limit broader applicability, though this is

acknowledged as a limitation.

**Author response:** Thanks, from bottom of our heart for reviewing our paper deeply andgiving constructive comments and suggestions. The limitation of the study mainly applicability part is described in the manuscript in clean and track changed type.

3. While proofreading was done, some minor grammatical or structural errors may persist.

**Author response:** Thanks, from bottom of our heart for reviewing our paper deeply andgiving constructive comments and suggestions. We ensure that we are also working on through the time of publication and we have also done some editing in manuscript also.

**Recommendation:**
Accept the Manuscript with Minor Revisions

The manuscript is well-structured, addresses an important public health issue, and has incorporated most reviewer feedback. The remaining concerns are minor and do not detract from the study's overall quality and contributions.

**Author response:** Thanks, from bottom of our heart for reviewing our paper deeply andgiving constructive comments and suggestions. We will keep in touch for any feedback regarding our research we ensure that we work on it anything needed, thanks again.

**Justification for Acceptance:**

- The study provides valuable insights into HPV vaccine uptake in a low-resource setting, with practical recommendations for improving vaccination rates.
- The mixed-methods approach strengthens the findings.
- Ethical and methodological rigor is demonstrated.
- The revisions have adequately addressed the reviewers' comments.
- Proceed with acceptance, pending these minor checks.

**Author response:** Thanks, from bottom of our heart for reviewing our paper deeply and giving constructive comments and suggestions. We will keep in touch for any feedback regarding our research we ensure that we work on it anything needed, thanks again.

**Final Decision:**

Accept with Minor Revisions

**Author response:** Thanks, from bottom of our heart for reviewing our paper deeply andgiving constructive comments and suggestions. We will keep in touch for any feedback regarding our research we ensure that we work on it anything needed, thanks again.
